# Bacterial Communities across Multiple Ecological Niches (Water, Sediment, Plastic, and Snail Gut) in Mangrove Habitats

**DOI:** 10.3390/microorganisms12081561

**Published:** 2024-07-30

**Authors:** Muna Al-Tarshi, Sergey Dobretsov, Mohammed Al-Belushi

**Affiliations:** 1 Marine Conservation Department, DG of Nature Conservation, Environment Authority, P.O. Box 323, Muscat 100, Oman; 2 Department of Marine Science and Fisheries, College of Agricultural and Marine Sciences, Sultan Qaboos University, Al Khoud 123 P.O. Box 34, Muscat 123, Oman; hamoodbeluch@gmail.com; 3 UNESCO Chair in Marine Biotechnology, Sultan Qaboos University, Al Khoud 123 P.O. Box 50, Muscat 123, Oman; 4 Central Laboratory for Food Safety, Food Safety and Quality Center, Ministry of Agricultural, Fisheries Wealth & Water Resources, P.O. Box 3094, Airport Central Post, Muscat 111, Oman

**Keywords:** plastic, mangrove, bacterial community, 16rDNA MiSeq, gut microbiota, Sea of Oman

## Abstract

Microbial composition across substrates in mangroves, particularly in the Middle East, remains unclear. This study characterized bacterial communities in sediment, water, *Terebralia palustris* snail guts, and plastic associated with *Avicennia marina* mangrove forests in two coastal lagoons in the Sea of Oman using 16S rDNA gene MiSeq sequencing. The genus *Vibrio* dominated all substrates except water. In the gut of snails, *Vibrio* is composed of 80–99% of all bacterial genera. The water samples showed a different pattern, with the genus *Sunxiuqinia* being dominant in both Sawadi (50.80%) and Qurum (49.29%) lagoons. There were significant differences in bacterial communities on different substrata, in particular plastic. Snail guts harbored the highest number of unique Operational Taxonomic Units (OTUs) in both lagoons, accounting for 30.97% OTUs in Sawadi and 28.91% OTUs in Qurum, compared to other substrates. Plastic in the polluted Sawadi lagoon with low salinity harbored distinct genera such as *Vibrio*, *Aestuariibacter*, *Zunongwangia*, and *Jeotgalibacillus*, which were absent in the Qurum lagoon with higher salinity and lower pollution. Sawadi lagoon exhibited higher species diversity in sediment and plastic substrates, while Qurum lagoon demonstrated lower species diversity. The principal component analysis (PCA) indicates that environmental factors such as salinity, pH, and nutrient levels significantly influence bacterial community composition across substrates. Variations in organic matter and potential anthropogenic influences, particularly from plastics, further shape bacterial communities. This study highlights the complex microbial communities in mangrove ecosystems, emphasizing the importance of considering multiple substrates in mangrove microbial ecology studies. The understanding of microbial dynamics and anthropogenic impacts is crucial for shaping effective conservation and management strategies in mangrove ecosystems, particularly in the face of environmental changes.

## 1. Introduction

In intertidal systems, the type and role of interactions among sediment microorganisms, animals, plants, and abiotic factors are complex and not well understood [1]. Such interactions are known to aid in nutrient provisioning and cycling, with the dynamics and interconnections being especially important in arid microtidal systems with limited nutrient influx [2]. Mangroves, coastal ecosystems prevalent in tropical and subtropical regions worldwide, span approximately 150,000 square kilometers across 123 countries [3]. Bacterial communities play important roles in marine ecosystems, contributing to nutrient cycling, degradation of organic matter, and maintenance of ecological balance [4]. According to a review of the literature, the primary compartments of mangrove systems are the rhizosphere, root systems, pneumatophores, bulk soil, water, sediments, and biota [5]. Understanding the distribution and composition of bacterial communities across diverse ecological niches is crucial for unraveling the complexities of microbial ecology and its implications for ecosystem health. Microorganisms in mangrove sediments and water, play an important role in nutrient cycling by facilitating the conversion and transportation of key elements [6]. Their importance is particularly evident in areas submerged beneath the tidal waterline, where they exhibit the greatest variety and abundance, benefiting the entire mangrove ecosystem [7]. Nevertheless, understanding of the intricate food webs and biogeochemical cycles in mangroves remains incomplete, primarily due to the limited information available on microbial species compositions and their ecology within these environments [8]. A diverse microbial community perpetually converts decaying vegetation into sources of nutrients, including nitrogen, phosphorus, and others, which are subsequently assimilated by plants [9]. In return, root exudates function as a nutrient source for microorganisms [10]. Hence, the significance of investigating microorganisms inhabiting root zones lies in elucidating various processes occurring in natural environments and the potential to manipulate these microbial populations for the benefit of the plant [11]. The emergence of metagenomics and next-generation sequencing technologies has allowed for a broader understanding of microbial diversity and functionality within mangrove ecosystems [12]. Given the ongoing risk of mangrove deforestation, understanding the composition of microbial communities associated with sediments, water, and organisms within mangrove habitats is critical for successful restoration efforts and assessing the health of mangrove ecosystems [13]. 

Investigating microorganisms within mangrove ecosystem sediment is essential for comprehending the distribution of bacterial communities, which play key roles in element cycling and fostering plant growth [14]. Furthermore, exploring microorganisms in mangrove ecosystems is crucial as mangrove rhizospheres harbor unique microbial assemblages that impact nutrient and sediment fluxes to the open sea [15]. These studies underscore the significance of understanding microbial dynamics in mangrove environments for elucidating their ecological functions and broader ecosystem processes. Previous studies indicate that the most dominant phylum associated with different substrata in mangroves is Proteobacteria [16]. 

Mangroves accumulate many different types of pollution including plastics and microplastics. The proliferation of marine plastic debris has emerged as a major global issue affecting oceanic ecosystems [17]. Plastics are colonized by different types of microorganisms, which form a unique community [18]. Such a microbial community is called a plastisphere [19]. The initial study on microbial colonization of plastic waste revealed that diatoms and certain species of Gram-negative bacteria had colonized polystyrene spherules discovered in the Sargasso Sea [20]. Members of the *Bacteroidetes* genus *Tunicatimonas*, which were originally isolated from sea anemones and later discovered to be prevalent on plastic debris in marine environments, are thought to have adaptations that allow them to exploit the novel niches created by plastic [21]. Zettler and colleagues [22] utilized next-generation sequencing and scanning electron microscope (SEM) to examine the microbial populations of plastic waste in the North Atlantic. They observed distinct differences between the bacterial communities on plastic debris and those present in the surrounding seawater. Meanwhile, a few plastic-degrading and hydrocarbon-degrading bacteria were found on the surface of plastic debris [23]. Wu with colleagues found that specific microorganisms, such as plastic-degrading bacteria and pathogens, were more abundant in plastics compared to water and sediment in the Haihe Estuary [24]. 

Gut microbiota refers to the microorganisms associated with the intestinal tract of animals, playing a vital role in physiological and biochemical reactions. Analyzing the gut microbiota within a mangrove environment offers a way to assess the extent of disturbance to mangrove ecosystems and evaluate the effects of anthropogenic pollutants [25]. In addition, studying the gut microbiota within mangrove systems is crucial due to the direct influence of environmental conditions on their microbial composition, highlighting the significance of conserving and sustainably managing estuaries [26]. This underscores the necessity of studying microbial communities in such ecosystems and implementing measures to preserve their ecological integrity. Studies have demonstrated that various environmental pollutants, including plastics, can impact the gut microbiota of different animals. Despite this significance, there is a scarcity of studies focusing on the gut microbiota of a gastropod mollusk *Terebralia palustris* (Linnaeus, 1767). This giant mangrove snail is commonly found in Omani mangrove lagoons, inhabiting mud and feeding on mangrove leaves, propagules, and seeds [27]. Given the importance of *T. palustris* in Oman’s mangrove ecosystems, investigating the microbes associated with this snail becomes crucial.

In Oman, mangroves consist of exclusively one species of *Avicennia marina* (Forssk.). The Ministry of Environment and Climate Affairs (now Environment Authority) launched in 2002 a campaign to expand the mangrove area, resulting in approximately 23.6 hectares now covered. This study was conducted in Sawadi lagoon (177 ha) which has the highest pollution and another natural mangrove lagoon (Qurum, 60 ha) was selected due to the lowest pollution [28]. This study aims to investigate the microbial communities inhabiting four distinct substrates within two mangrove lagoons located in Oman: Sawadi and Qurum. Specifically, our objectives are twofold: (1) Characterize the microbial communities present in sediment, surface water, plastic debris, and the gut of *T. palustris* snails within these mangrove lagoons, and (2) Compare the composition and diversity of bacterial communities across the aforementioned substrates and between the two mangrove lagoons using MiSeq sequencing of the 16S rDNA gene.

## 2. Materials and Methods

### 2.1. Locations and Site Characteristics

Sampling activities were conducted from July 15th to 20th, 2023, in two lagoons located in the Sea of Oman, Oman. These lagoons, Sawadi (23°45′41.99″ N 57°47′29.64″ E) and Qurum (N 23°37′20.45″/E 58°28′37.34″) exhibit distinct mangrove vegetation characteristics (Supplementary Table S1). Each lagoon was divided into three transects (T1, T2, and T3), representing the seaward fringe, inside the forest, and landward fringe, respectively. Detailed design schematics can be found in the Supplementary Materials (Supplementary Table S1). During the study period, samples of sediment, water, snails, and plastics were collected from each Sawadi and Qurum lagoon transects. The sampling protocol followed methodologies outlined by [24] with necessary adaptations. Notably, all samples were collected concurrently. Diverse physical attributes were assessed at the sampling sites. Specifically, water parameters such as dissolved oxygen levels, conductivity, salinity, pH, and temperature were measured using the calibration method of portable water quality meters (SevenGoTM SG3, Japan) (Supplementary Table S2).

### 2.2. Sediments Collection

In each sampling site (Sawadi and Qurum), three samples from the upper layer (depth = 0–5 cm) of sediments were collected in a quadrat 30 cm × 30 cm based on the previously described methodology [29,30]. Only one sample was taken from each transect, representing each zone. Briefly, samples of surface sediment were collected using a metal spoon. About 400 g of sediment per sample was carefully gathered and placed in separate sterile 500 mL glass containers. Samples were transported to the laboratory immediately on ice. Upon arrival at the laboratory, all samples were stored in a lab freezer at −80 °C until further analysis.

### 2.3. Water Collection 

In each sampling site (Sawadi and Qurum) three samples of water were collected. Only one sample was taken from each transect, representing each zone. Two liters of water samples were taken from a depth of 30 cm below the surface [24]. The collected water samples underwent a two-step filtration process to eliminate potential contaminants. First, they were passed through quantitative stainless steel tower sieves with a pore size ranging from 60 µm to 500 µm. This step removed any impurities from the samples. Subsequently, the filtered water samples were subjected to a secondary filtration using 0.2 µm sterile Millipore cellulose acetate membranes (Whatman, Cytiva, Marlborough, MA, USA). This additional filtration step ensured the removal of even finer particles. Then, the filters were cut into smaller pieces in preparation for the DNA extraction protocol [31].

### 2.4. Plastics Collection and Characterization

Plastic samples prevalent in the lagoons were collected using sterile stainless forceps from the surface sediments within a 100 m^2^ quadrat in each transect (T 1–3). Various types of the most prevalent plastics, differing in size and color were collected from three quadrats at each lagoon. In the laboratory, the plastic samples underwent a triple rinse with distilled DNA-free water (Sigma-Aldrich, Darmstadt, Germany) and were subsequently stored in 250 mL sterile screw-neck glass tubes and kept at −80 °C until further analysis (see below). 

The color, shape, and size of plastics were recorded during the sampling process (see Supplementary Table S4). The types of polymers were identified using Attenuated Total Reflection Fourier Transformed Infrared Spectroscopy (ATR-FTIR) with the ATR-MIRacle^TM^ PIKE instrument (Agilent Technologies, Santa Clara, CA, USA). The detector’s spectral range covered 400 to 4000 cm^−1^, with a resolution of 8 cm^−1^ for 16 scans. The spectra were processed using (MicroLab software, Version 6.2.18) and compared with standard polymer spectra to facilitate analysis (Supplementary Figure S1).

### 2.5. Snail Collection

Ten *Terebralia palustris* snails were randomly collected within 100 m^2^ from both Sawadi and Qurum lagoons. To maintain snail integrity for further examination, they were promptly chilled on ice. In the laboratory, snail shells were crushed utilizing a vice, followed by the dissection of their guts using a sterile scalpel. Each snail gut was then delicately placed into a sterile 250 mL glass container and preserved at −80 °C for subsequent analysis.

### 2.6. Isolation of DNA

DNA from water, sediments, plastics, and snail gut samples were extracted using the Powersoil DNA extraction kit (Qiagen, Hilden, Germany). For this procedure, 250 mg of sediment, 250 mg of cut filter, 250 mg of plastic, and 250 mg of snail gut were added to separate tubes. Before extraction, large pieces were cut into micro-sized fragments using sterilized scissors. DNA extraction was carried out according to the instructions provided in the manual. From each location, DNA from 3 samples of sediment, 3 samples of water, 3 samples of plastic, and 10 guts of snails was obtained. To ensure quality control, DNA-free water was utilized instead of the sample during DNA extraction to detect any potential contamination. Following extraction, the quality and quantity of the extracted DNA were evaluated using a NanoDrop™ Lite spectrophotometer (Thermo Fisher Scientific, Waltham, MA, USA). In the case of low quality and quantity of DNA, it was re-extracted from new samples collected earlier.

### 2.7. DNA Sequencing and Identification of Bacteria

The DNA samples obtained from sediments, water, the gut of snails, and plastic underwent Illumina MiSeq paired-end sequencing (2 × 300  bp) at Molecular Research (MRDNA) in Texas, USA. Before sequencing, PCR was conducted with universal primers targeting the bacterial hypervariable regions V3–V4 of the 16S rRNA gene (515 F: 5′-GTGCCAGCMGCCGCGGTAA-3′ and 806 R: 5′-GGGACTACHVGGTWTCTAAT-3′), following specific PCR conditions. The subsequent Illumina MiSeq paired-end sequencing process involved several data processing steps. These steps included the removal of barcode and primer sequences, merging reads to generate Raw Tags using Vsearch v2.4.4, filtering out low-quality reads, and defining OTUs based on 97% sequence identity. Representative sequences for OTUs were annotated using the SILVA12.8 databases, with validation from RDPII and NCBI databases. The relative abundance in the percentage of each bacterial group in each sample was calculated.

### 2.8. Data Analysis and Statistical Methods

In this study, descriptive statistics were computed for OTUs to characterize their distribution in sediment samples from Sawadi and Qurum lagoons. Additionally, relative abundances at the phylum, class, and genus levels were analyzed across various substrates and microbial communities. For phyla and genera data with an abundance greater than 1% was analyzed, while for class level, there was no cut-off. Normality tests, specifically the Shapiro-Wilk test, were conducted to assess the distribution of taxonomic data, followed by Kruskal-Wallis tests to detect significant differences in taxonomic composition. Subsequently, Dunn Post Hoc tests were employed for pairwise comparisons following significant findings in the Kruskal-Wallis tests, aimed at identifying specific pairs of substrates or microbial communities exhibiting significant differences in taxonomic composition. Through these analyses, the study aimed to elucidate the diversity and taxonomic structure of microbial communities in the lagoons and discern potential differences.

Data analysis includes the calculation of the percentage of relative abundances, mean, and standard deviation numbers in each bacterial group. Principle Component Analysis (PCA) was employed to analyze differences in bacterial communities across sampling sites and substrates. Shannon diversity indices, evenness index, and Chao indices were calculated to estimate genus richness. Shared and unique OTUs among different substrates were plotted using Venn diagram (https://cir.nii.ac.jp/all?q=http://bioinfogp.cnb.csic.es/tools/venny/index.html, accessed on 23 May 2024). The data were plotted using OriginPro software, version 2023b and PAST software, version 1.0.0.0.

## 3. Results 

### 3.1. Chemical Composition of Plastics

Polymer analysis via FTIR revealed the presence of Polyethylene (PE), Polypropylene (PP), Polystyrene (PS), and High-Density Polyethylene (HDPE) among the collected plastics (Supplementary Figure S1). 

### 3.2. Diversity of the Bacterial Communities 

Bacterial 16S rDNA gene amplicon sequencing conducted in two lagoons, Sawadi and Qurum, encompassing sediment, water, snail guts, and plastics, yielded a total of 2,000,189,234 sequences with lengths ranging from 230 to 250 bp. Diversity indices provided in Supplementary Tables S3 and S4 were calculated based on genera abundance within each substratum across the two lagoons. Upon comparing diversity indices among substrates within each lagoon, distinct patterns emerged. In Sawadi Lagoon (A), sediment and plastic substrates displayed higher Shannon index values than water and gut samples, suggesting greater species diversity. Additionally, sediment and plastic substrates exhibited relatively even distributions of genera, as indicated by their evenness index values, while water and gut samples showed lower evenness, implying a less uniform distribution. Furthermore, Chao index values were higher for sediment and plastic substrates compared to water and gut samples, implying potentially greater genus richness in sediment and plastic substrates. 

In Qurum Lagoon, Shannon index values were generally lower across all substrates compared to Sawadi, indicating lower species diversity (Supplementary Tables S3 and S4). However, evenness index values suggested relatively uniform distributions of genera across sediment, water, and plastic substrates, while gut microbes exhibited lower evenness. Similarly, Chao index values were higher for communities on sediment and plastic substrates compared to ones associated with water and gut samples, suggesting potentially higher genus richness in sediment and plastic substrates, similar to observations in Sawadi.

### 3.3. Abundance of Bacterial Communities

#### 3.3.1. OTUs from All Substrates in Two Lagoons 

In Sawadi and Qurum lagoons, a total of 227,553 OTUs were detected across all four substrates (sediments, water, snail guts, and plastic). In Sawadi, the total number of OTUs was 111,631, whereas in Qurum, it reached around 115,922. Among the environments sampled in Sawadi, the gut of snails exhibited the highest OTU count (34,583), while both water and plastic substrates displayed relatively similar OTU counts, totaling 28,887 and 28,307, respectively. In Qurum, a similar pattern emerged, with the gut of snails having the highest OTU count at 33,516. The water and plastic substrates in Qurum also demonstrated comparable OTU counts, with 29,782 and 29,618, respectively, whereas sediment exhibited the lowest OTU count at 23,005.

**Figure 1:**
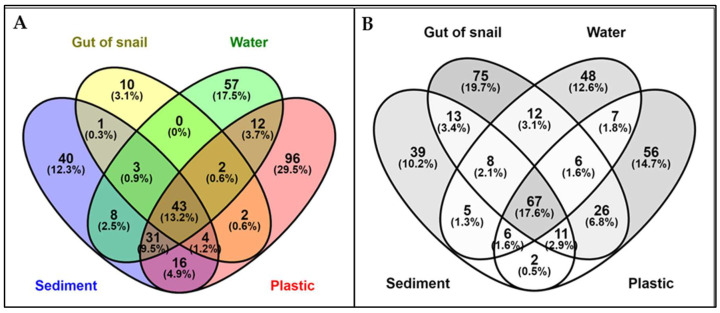
Venn diagrams showing counts and percentages of common and unique OTUs in microbial communities across four substrates in Sawadi (**A**) and Qurum (**B**) lagoons.

The Venn diagrams show the distribution of unique and shared OTUs among four substrates (water, sediment, snail gut, and plastic) in two lagoons, Sawadi and Qurum (Figure 1). In Sawadi lagoon (Figure 1A), plastic harbors the highest proportion of unique OTUs (29.5%), followed by water (17.5%) and sediment (12.3%). Conversely, the snail gut microbiome exhibits the lowest percentage of unique OTUs (3.1%). Approximately 13% of OTUs are shared among all four substrates. Most OTUs (9.5%) are shared between communities in sediment, water, and plastic. A total of 4.9% of OTUs are shared among microbes in sediments, snail guts, and plastics. In contrast, in the Qurum lagoon (Figure 1B), snail gut microbes host the highest proportion of unique OTUs (19.7%), followed by plastic (14.7%), water (12.6%), and sediment (10.2%). Approximately 18% of OTUs are shared among all substrates. The shared OTUs between sediment, snail gut, and plastic communities are 2.9%, while those between sediment, snail gut, and water communities are 2.1%. In terms of pairwise comparisons between microbes on two substrates, the shared OTUs between water and plastic are the most abundant in both lagoons, with 6.8% in Qurum and 3.7% in Sawadi (Figure 1). Furthermore, in Sawadi lagoon, there are no shared OTUs between water and snail gut microbiota, while in the Qurum lagoon, only 0.5% of OTUs are shared between sediment and plastic communities.

#### 3.3.2. OTUs from Different Substrates in Two Lagoons

**Figure 2:**
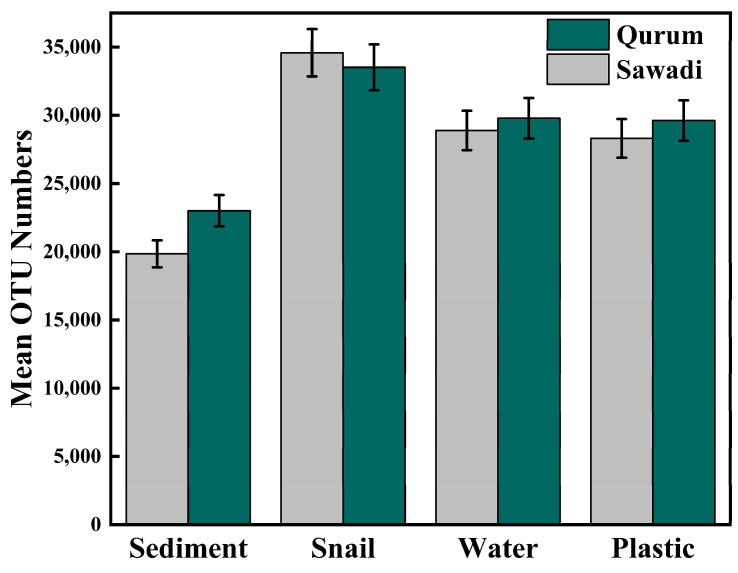
Mean abundance of OTUs in four substrates (sediment, gut, water, and plastic) from Sawadi and Qurum mangrove sites in the Sea of Oman. Data are the mean ± standard deviation.

There was a significant variation in the amount of OTUs between microbes on different substrates and in different lagoons suggesting spatial variability of microbes (ANOVA, *p* = 0001) (Figure 2; Supplementary Table S6). The lowest amount of OTUs was recorded for bacteria associated with sediment samples, while the highest one was found in snail guts (Figure 2; Supplementary Table S4). When considering plastic communities, notably high OTU counts were observed in the Q3T3 transect in the landward fringe (32,628) and the S1T1 transect in the seaward fringe (31,226). This indicates a richer diversity of bacterial communities in these specific areas compared to others. In contrast, bacterial communities in water demonstrate relatively consistent OTU counts ranging from 29,543 to 30,072 across all locations, suggesting a relatively stable and comparable bacterial diversity in the water among the sampled locations (Supplementary Figure S2). However, the sediment samples display more variability in OTU counts. For instance, the S1T1 transect in the seaward fringe exhibits the lowest OUT counts (16,917), while the Q3T3 transect in the landward fringe (25,949) and Q1T1 transect in the seaward fringe (28,003) display higher OTU counts, indicating a more diverse bacterial community in these particular locations.

The distribution of OTUs in both lagoons was found to deviate from normal according to the Shapiro–Wilk test (Sawadi: W = 0.94473, *p* = 0.68335; Qurum: W = 0.91719, *p* = 0.52131). Furthermore, ANOVA results indicated a substantial difference in OTU distribution between the two lagoons (F (1, 9) = 57.88345, *p* < 0.0001). 

### 3.4. Taxonomic Analysis of Bacterial Communities 

#### 3.4.1. Distribution and Relative Abundance of Bacterial Communities across Locations and Substrates: Phylum-Level

**Figure 3:**
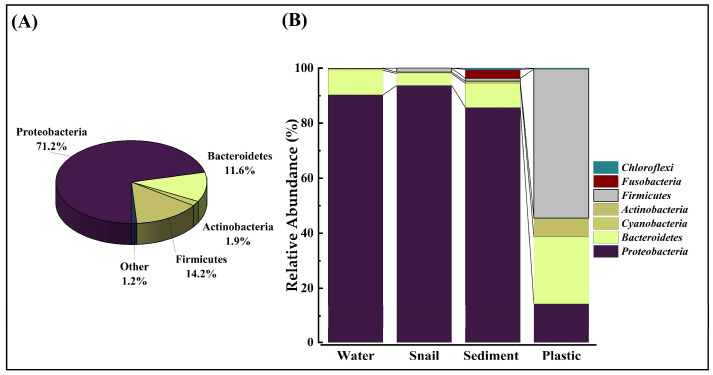
Relative abundance of bacterial phyla. The pie chart represents the relative abundance of phyla in percentage (**A**) and (**B**) the mean ± SD relative abundance of bacterial phyla in water, snail guts, sediments, and plastic.

Based on the overall phylogenetic analysis results, the sequence analysis of bacterial communities showed that Phylum Proteobacteria is the most dominant in microbial communities (71%, Figure 3A). This is followed by Phyla Firmicutes (14%) and Bacteroidetes (12%). The phylum Actinobacteria (2%) had the lowest abundance (Figure 3A). 

Figure 3B demonstrates the relative abundances of different bacterial phyla across sediment, water, gut microbiota, and plastic. Similar bacterial phyla were found in water, sediments, and gut microbiota samples dominated by Proteobacteria. However, completely different phyla were observed for plastic debris (Figure 3B), where the dominant phyla were Firmicutes and Bacteroidetes.

A non-normal distribution was confirmed for all substrates in both lagoons (sediment: *p* < 0.0001; water: *p* < 0.0001; gut: *p* < 0.0001; plastic: *p* = 0.01 in Sawadi and *p* = 0.002 in Qurum). Nonetheless, Kruskal-Wallis tests revealed no significant difference in median abundances between Sawadi and Qurum for sediment (*p* = 0.4057), water (*p* = 0.8477), and plastic (*p* = 0.4942) communities at a phyla-level. However, a noteworthy difference in median abundance was observed in the gut microbiota (*p* = 0.02535), with post hoc analysis indicating a discernible difference between the gut microbial communities of *T. palustris* snails in Sawadi and Qurum lagoons at the phyla-level (*p* = 0.02525) (Supplementary Tables S5–S10).

#### 3.4.2. Distribution and Relative Abundance of Bacterial Communities across Locations and Substrates: Class-Level

**Figure 4:**
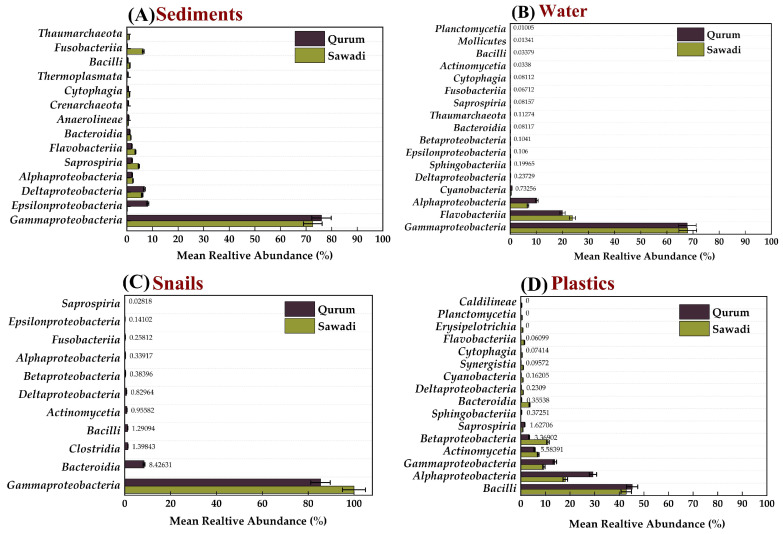
Mean relative abundance of classes across four substrates in two lagoons. Sediment (**A**), water (**B**), snail guts (**C**), and plastic (**D**).

At the class level, Gammaproteobacteria dominated the sediment, gut of snails, and water microbiota, comprising 71.35%, 92.66%, and 67.97% respectively. The composition of bacteria on different substrates varied at the class level (Figure 4). Water samples exhibited the presence of class Bacteroidia, although in low abundance compared to other substrates. Additionally, class Bacilli was unique to plastic, constituting approximately 44.06% compared to other substrates. Some classes were exclusively found in plastic and absent in other substrates, including Erysipelotrichia, Sphingobacteriia, Planctomycetia, Caldilineae, and Synergistia (Figure 4).

The examination of relative abundances of different classes across sediment, gut microbiota, water, and plastic debris within Sawadi and Qurum lagoons is shown in Figure 4. In sediment samples, a non-normal distribution was evident in both lagoons, as indicated by Shapiro-Wilk tests (Sawadi: W = 0.3922, *p* = 9.45 × 10^−7^; Qurum: W = 0.3859, *p* = 8.55 × 10^−7^). However, no significant difference in the median abundance of bacterial classes between the two lagoons was observed (*p* = 0.7828) (Supplementary Tables S11–S15). For gut microbiota samples, non-normal distribution was found in both lagoons (Sawadi: W = 0.3657, *p* = 1.00 × 10^−7^; Qurum: W = 0.3819, *p* = 1.54 × 10^−7^). Importantly, a significant difference in the median abundance between microbial classes in the Sawadi and Qurum lagoons was detected (*p* = 0.001524). Snail gut microbiota in Sawadi showed distinct characteristics compared to Qurum snail gut microbiota. In water samples, a non-normal distribution of relative abundances was observed in both lagoons (Sawadi: W = 0.4054, *p* = 2.25 × 10^−7^; Qurum: W = 0.4093, *p* =2.41 × 10^−7^). However, no significant difference in the median abundance of classes between the Sawadi and Qurum lagoons was observed (*p* = 0.6919). Similarly, non-normal distribution was found in class relative abundances in plastic samples from both lagoons (Sawadi: W = 0.594, *p* =1.42 × 10^−5^; Qurum: W = 0.565, *p* = 7.81 × 10^−5^). Nevertheless, a significant difference in median abundance between the two lagoons was detected (*p* = 0.04981) (Supplementary Tables S11–S15).

#### 3.4.3. Distribution and Relative Abundance of Bacterial Communities across Locations and Substrates: Genus-Level

**Figure 5:**
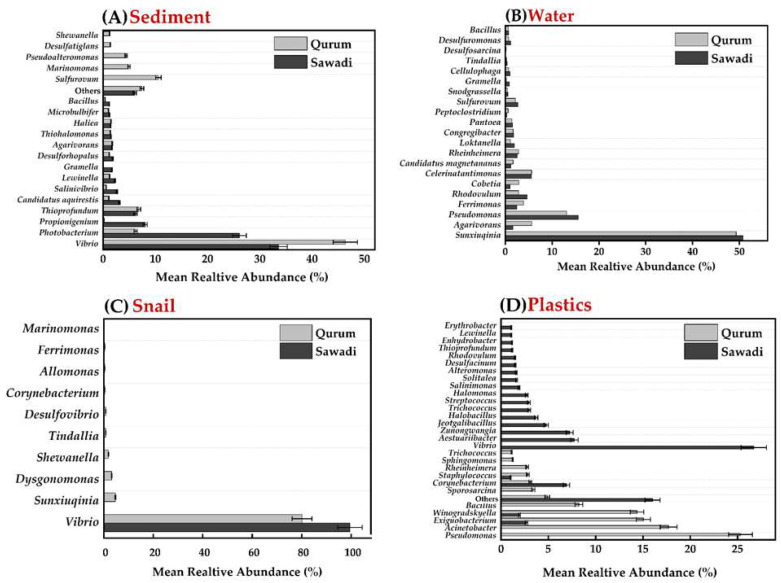
Mean relative abundance of genera across four substrates in two lagoons. Sediment (**A**), water (**B**), snail guts (**C**), and plastic (**D**).

The study of bacterial communities across different substrates found no significant variation in genus abundance between locations (*p*-value = 0.9298) Figure 5. However, there was a significant variation in the dominant genera between substrata, except in the gut of snails (*p*-value = 0.007051) (Figure 5C). In the sediment of the Sawadi and Qurum lagoons, the genus *Vibrio* was found to be the most abundant, accounting for 33.59% and 46.36% of the bacterial communities, respectively. Other notable genera in the sediment of Sawadi included *Photobacterium* (26.11%) and *Propionigenium* (8.03%), whereas, in Qurum, *Sulfurovum* (10.59%) and *Thioprofundum* (6.85%) were also found (Figure 5A). In the gut of snails, *Vibrio* was overwhelmingly dominant in both locations, with a relative abundance of 99.56% in Sawadi and 80.15% in Qurum (Figure 5C). Other genera present in significantly smaller proportions included *Shewanella* (0.12%) and *Marinomonas* (0.12%) in Sawadi, and *Sunxiuqinia* (4.68%), and *Dysgonomonas* (3.17%) in Qurum.

The water samples showed a different pattern, with *Sunxiuqinia* being the dominant genus in both Sawadi (50.80%) and Qurum (49.29%) (Figure 5B). In addition to *Sunxiuqinia*, *Pseudomonas* was also prevalent in both locations, making up 15.60% of the community in Sawadi and 13.08% in Qurum. Other significant genera included *Celerinatantimonas* (5.58%) in Sawadi and *Agarivorans* (5.67%) in Qurum.

On plastic surfaces, the bacterial communities differed significantly between the two lagoons (Figure 5D). In Sawadi, *Vibrio* was the most dominant genus (26.72%), followed by *Aestuariibacter* (7.76%) and *Zunongwangia* (7.26%). Conversely, in Qurum, *Pseudomonas* dominated the plastic surfaces with a relative abundance of 25.30%, and other notable genera included *Acinetobacter* (17.71%) and *Exiguobacterium* (15.05%).

The comparative analysis of genus relative abundance between Sawadi and Qurum lagoons yielded noteworthy observations. For the genus-level analysis in plastic samples, non-normal distribution was evident in both lagoons, as indicated by the Shapiro–Wilk test (Sawadi: W = 0.59906, *p* < 0.0001; Qurum: W = 0.60996, *p* < 0.0001). Despite this, no significant difference in median abundance between the relative abundance of bacterial genera in the two lagoons was detected (p = 0.1294). Similarly, in sediment samples, non-normal distribution was observed in both lagoons (Sawadi: W = 0.5655, *p* =1.32 × 10^−6^; Qurum: W = 0.4626, *p* = 1.48 × 10^−7^). However, there was no significant difference in median abundance between bacterial genera in Sawadi and Qurum lagoons (*p* = 0.9784). In water samples, non-normal distribution was present in both lagoons (Sawadi: W = 0.4065, *p* = 3.11 × 10^−8^; Qurum: W = 0.4199, *p* = 4.03 × 10^−8^), with no significant difference in median abundance between the two lagoons (*p* = 0.9298). Conversely, for gut microbiota samples, non-normal distribution was observed in both lagoons (Sawadi: W = 0.3667, *p* = 1.03 × 10^−7^; Qurum: W = 0.4143, *p* = 3.65 × 10^−7^). Importantly, a significant difference in median abundance between Sawadi and Qurum lagoons was detected (*p* = 0.007051), highlighting distinct characteristics in the gut microbiota of *T. palustris* snails between the two lagoons (Supplementary Tables S16–S20). 

### 3.5. Structure of Bacterial Communities

**Figure 6:**
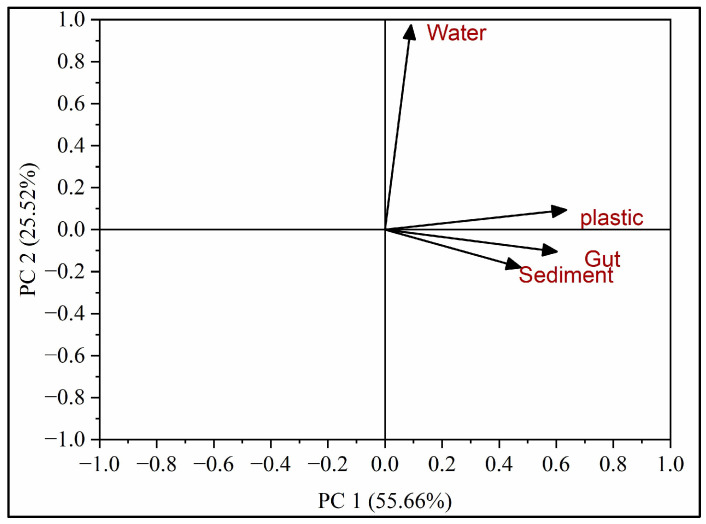
Principal component analysis (PCA) of OTUs at different substrates (water, sediments, snail gut, and plastics).

The structure of bacterial communities was analyzed by the principal component analysis. The PCA identified two main axes of variation, known as PC1 and PC2 which explained more than 80% of variation (Figure 6). Principal Component 1 (PC1 represented a significant portion (55.66%) of the total variability in the OTUs. PC1 captured the most important sources of variation among the bacterial communities. The strong positive correlations observed between PC1 and the four substrates (snail gut, sediment, water, and plastic) suggest that these substrates play a significant role in shaping the variability seen in the bacterial communities. This implies that there are noticeable differences in both the composition and abundance of bacterial species across these substrates. Principal Component 2 (PC2) accounted for a considerable portion (25.25%) of the observed variability in the bacterial communities (Figure 6). While PC1 primarily captured the main sources of variation, PC2 represents additional, although less pronounced, differences among the bacterial communities across the substrates. The specific factors influencing the variation along PC2 may differ from those affecting PC1 and could reflect unique ecological factors, like salinity, pH, nutrients, etc., or other environmental factors.

## 4. Discussion 

Mangrove forests are vital ecosystems found in tropical and subtropical coastal areas worldwide [32]. Within these forests, microorganisms, like bacteria and fungi, play a big role in maintaining their health by recycling important nutrients [33]. They are crucial for mangrove productivity, conservation, and recovery [34]. Bacteria and fungi make up most of the biomass here [35] and bacteria play a key role in various cycles that regulate energy flow in mangrove habitats [36]. However, there is limited information about the composition of bacterial communities associated with different substrata in mangrove habitats. 

### 4.1. Diversity of Microbes between Two Lagoons and across Different Substrates in the Study Area

The comparative analysis of microbial diversity across various substrates in Sawadi and Qurum lagoons highlights the complexity of microbial ecosystems in different environments. The similarities in microbial abundance between Sawadi and Qurum for water, sediment, plastic and gut-associated microbial communities indicate that similar environmental conditions influence microbial colonization in both lagoons. Specific examples of these environmental conditions include temperature ranges, which are 30.7 °C to 32.2 °C in Sawadi and 30.8 °C to 32 °C in Qurum, creating similar thermal environments conducive to microbial growth [37]. The salinity levels are also comparable, with Sawadi ranging from 35.9 to 36.2 and Qurum from 37.3 to 37.9, providing a stable osmotic environment for microbial communities [37,38]. Additionally, total dissolved solids (TDS) levels are close, with Sawadi having values between 27.1 and 27.3, and Qurum between 28.1 and 28.5, indicating similar concentrations of minerals and organic matter essential for microbial metabolic processes [37,38,39]. Both lagoons have similar electrical conductivity values, reflecting the overall ionic content of the water, with Sawadi ranging from 54.2 to 54.7 μs /cm and Qurum from 56.2 to 57.1 μS/cm, supporting a stable ionic environment for microbial communities [40]. The refractive index values are also close, with Sawadi ranging from 18.30 to 18.50 and Qurum from 17.50 to 17.80, indicating similar water density and composition in both lagoons Supplementary Table S2.

The significant difference in microbial communities in the gut of snails between the two locations, Qurum and Sawadi, can be attributed to several environmental and ecological factors influenced by the differing conditions of each lagoon [41]. Qurum Lagoon features natural, old mangroves [27], which provide a stable and mature ecosystem with established ecological interactions [42]. Natural mangroves often have complex root structures and rich organic matter that support diverse microbial communities [43]. In contrast, Sawadi Lagoon is a man-made mangrove forest [27], which might lack the complexity and stability of natural mangroves [27]. The relatively younger and artificial environment could lead to different microbial colonization patterns and community structures [44].

Additionally, pollution levels play a crucial role. As a marine protected area, Qurum likely experiences lower levels of pollution and human disturbance, supporting a more balanced and diverse microbial ecosystem. On the other hand, Sawadi is noted for being polluted by marine litter and microplastics [28]. Pollution, especially microplastics, can disrupt microbial communities by introducing new surfaces for colonization, altering nutrient cycles, and potentially introducing harmful substances that affect microbial survival and interactions [45].

Environmental conditions such as water quality and sediment composition further influence these differences. Variations in water quality parameters like salinity, nutrient levels, and the presence of contaminants can significantly impact microbial community composition [46]. Moreover, the type and quality of sediments, influenced by natural versus man-made environments, affect the availability of habitats and nutrients for microbes [47].

Host-microbe interactions also play a role. Snails in different environments may ingest different types and amounts of microbes from their surroundings, influenced by the quality and type of organic matter available in natural versus polluted mangroves [38]. Snails in polluted environments like Sawadi might experience more stress, which can alter their gut microbiota [48]. Stress can affect the immune response of snails, making their gut environment more hospitable to certain microbes and less to others [49].

The significant difference in microbial communities on plastic in Qurum and Sawadi lagoons stems from their environmental conditions and ecological contexts. Qurum, a marine protected area with natural, old mangroves, offers stability and lower pollution levels, supporting a diverse microbial community on plastic [50]. In contrast, Sawadi, with man-made mangroves and higher pollution levels, including microplastics, presents a more disturbed environment favoring different microbial communities on plastic. Water quality, sediment composition, and pollution levels influence microbial colonization, resulting in distinct microbial profiles on plastic between the two lagoons [51].

The variations in microbial diversity across different substrates and lagoons can be explained by several factors, including substrate composition, environmental conditions, and microbial interactions [52]. For example, sediment environments tend to provide a wide range of habitats and nutrients, leading to higher microbial diversity compared to substrates like water [53]. The differences in water microbial diversity between Sawadi and Qurum could be due to variations in factors such as water temperature, salinity, and nutrient availability, which directly impact the composition of microbial communities [54] Supplementary Table S2.

The remarkably low diversity of gut microbiota in snails across both lagoons shows a specialized microbial community within the snail digestive system. This specialization likely arises from the unique physiological and nutritional needs of snails, which favor specific microbial taxa capable of thriving in this environment [55].

Despite being artificial, plastic surfaces support relatively high microbial diversity, possibly because of the accumulation of organic matter and the formation of biofilms [56]. The slight variations in microbial diversity between Sawadi and Qurum plastic samples may indicate differences in environmental conditions or microbial colonization dynamics on these surfaces Supplementary Table S3.

When comparing between lagoons, slight differences in microbial community structure emerge, as shown by metrics like the Chao index. These differences may be influenced by local environmental conditions, human activities, or geographical factors unique to each lagoon. Considering such location-specific factors is essential in microbial diversity studies.

### 4.2. Dominant Groups of Bacteria 

In both Sawadi and Qurum lagoons, Proteobacteria was the most abundant phylum. This matches findings from similar studies, even though they focused on different substrates [57]. For example, Proteobacteria emerged as the most prevalent group in mangrove sediments in several related studies [58,59]. Additionally, research revealed Proteobacteria to be dominant in the surface water of mangroves [60], and the gut of snails in Chinese mangroves [61]. Moreover, Proteobacteria were found to be dominant in various types of plastic polymers [62]. Among the phylum Proteobacteria, Gammaproteobacteria was the most dominant class in all investigated substrata in our study. Previous studies reported that the increased prevalence of the classes Gammaproteobacteria and Deltaproteobacteria, contributed to the detoxification of pollutants in mangrove soils [63]. This could indicate the potential role of these microbes in mangrove habitats. 

Bacteroidetes emerged as the second most abundant phylum of bacteria present in all substrata in both lagoons. Several researchers have reported higher relative abundances of Bacteroidetes in various environments including mangroves [64]. This phylum holds significant ecological importance due to its vital roles in carbon, nitrogen, and sulfur cycling [65]. The higher abundance of Bacteroidetes on plastic compared to surface water, sediments, and gut of snails in the same locations suggests their adaptability to colonize different surfaces and substrata [66].

*Vibrio* was the most dominant genus of bacteria in all sampled locations and substrata except water samples. The genus *Vibrio* encompasses over 100 species, with numerous ones establishing relationships, whether symbiotic or pathogenic, with marine organisms like fish, mollusks, and crustaceans [67]. This genera was found in the gut of snails [68], and the surface of plastics, especially in areas with elevated nutrient levels and reduced salinity [69]. Although not all *Vibrio* species are pathogenic, some of them, like *V. parahaemoliticus, V. vulniferus*, and *V. cholerae* could cause diseases [70]. This could suggest that potentially pathogenic species are present in mangrove habitats which could be studied in the future. 

### 4.3. Bacterial Communities on Different Substrates

We hypothesized that bacterial communities associated with sediment, plastic, water, and the gut of snails in mangroves would be different. This could be due to differences in the physical and chemical characteristics of the tested substrates [71]. In comparison with other substrata, the water samples exhibited the presence of genera such as *Sunxiuqinia* and *Pseudomonas*. The genus *Sunxiuqinia* belongs to the order Bacteroidales and contains anaerobic heterotrophic bacteria isolated from marine sediments [72]. The genus *Pseudomonas* is one of the common Gammaproteobacteria widely present in sediments [73], water [74].

This was the first study of gut microbiota of *T. palustris* snails which dominated mangrove habitats in Oman. This snail is feeding on mangrove leaves, propagules, and seeds [27]. Our research found that gut microbiota is dominated by Gammaproteobacteria, manily *Vibrio* sp. Other studies demonstrate the dominance of Proteobacteria in guts of vivaparid snails [75] and freshwater snails [76]. However, in these studies other genera of bacteria, like *Lelliottia*, *Clostridium* and *Bacillus* were dominant. The differences in dominant species could be explained by the diet of snails [77]. The bacterium *Vibrio algivorus* was isolated from the gut of the marine snail *Turbo cornutus* [78]. Similarly, a number of *Vibrio* species were isolated from the digestive tube of the mud snail *Bullacta exarata* [79]. While some authors pointed out that gut-associated microbes could be responsible for the degradation of plastics [80] and cellulose [81], there is no evidence of these functions of *Vibrio* spp. 

Bacteria on plastics were different and dominated by phyla Firmicutes and Bacteroidetes and to a lesser extent by Actinobacteria. Bacterial classes Erysipelotrichia, Sphingobacteriia, Planctomycetia, Caldilineae, and Synergistia were found exclusively on plastics. This could be due to the unique physical and chemical properties of plastics favoring the growth and colonization of certain bacteria [82]. It is known that Actinobacteria are involved in substance recycling, the degradation of complex polymers, the production of bioactive molecules [83], and the bioremediation of pesticides and heavy metals [84]. Another dominant phyla Firmicutes are responsible for organic degradation in biodegradable plastics like Poly(p-dioxanone) PPDO [85] and the ability to utilize xylose from plastics as a carbon source [86].

In our study, bacterial genera *Aestuariibacter*, *Pseudomonas*, *Zunongwangi*, and *Exiguobacterium* were dominant on plastic substrata. Previous studies suggested that *Aestuariibacter* and *Pseudomonas* were among the top 9 plastic-degrading genera [87]. *Aestuariibacter* was the most dominant bacteria on weathered polyethylene in the marine environment [88]. The genus *Zunongwangia* was identified as one of a few chlorinated paraffin-degraders from marine sediments. *Exiguobacterium* bacteria could degrade a variety of plastics including polystyrene [89,90], low-density polyethylene [91], and polypropylene [92]. These examples suggest that some bacteria found on plastic in our study can be responsible for plastic biodegradation. 

### 4.4. Bacterial Communities in Different Lagoons

In most cases, microbial communities associated with different lagoons were different. For example, class Fusobacteria were prominently present in the sediment of Sawadi lagoon, while they were entirely absent in the sediment of Qurum. Similarly, genera *Vibrio*, *Aestuariibacter*, *Zunongwangia*, *Jeotgalibacillus*, *Halobacillus*, and *Trichococcus* were dominant in plastic samples from Sawadi lagoon, while *Pseudomonas*, *Acinetobacter*, *Exiguobacterium*, *Bacillus*, and *Sporosarcina* genera were found in Qurum lagoon. Additionally, snail gut microbiota in Sawadi showed distinct characteristics compared to snail gut microbiota in Qurum. The differences between microbial communities in Qurum and Sawadi lagoons could be attributed to the differences in environmental conditions. Sawadi waters have lower salinity than in Qurum. This could promote the growth of bacteria in Sawadi adapted to lower salinity. This is further supported by the Principal Component Analysis (PCA) which shows that environmental conditions, such as salinity, pH, and nutrient levels as well as the specificity of the substrate, play a significant role in shaping bacterial community composition. Previous studies demonstrated that salinity significantly impacted nitrogen cycling in mangrove communities [93] and the structure of their microbial community [94]. Additionally, the level of environmental pollution could impact the composition of microbial communities. Our previous study suggested that Qurum mangroves showed the lowest density of marine litter and microplastic pollution among seven studied mangrove habitats in the Sultanate of Oman [94]. In opposite, Sawadi had the highest level of microplastic pollution. Marine pollution could result in anoxia and stress on mangrove trees, which could affect the whole mangrove ecosystem. This fact could explain the dominance of bacteria adapted to high levels of pollution, sediment contamination, and lower oxygen concentrations [95]. Similarly, it was demonstrated that bacterial communities in contaminated mangroves had reduced nitrogen fixation but enhanced heavy metal tolerance, sulfate reduction and methanogenesis [96]. The factors contributing to the differences between microbial communities on the same substrate in Sawadi and Qurum lagoons remain unclear and should be investigated in future studies. 

## 5. Conclusions

This study aimed to characterize and compare the microbial communities present in sediment, surface water, plastic debris, and the gut of *T. palustris* snails within two mangrove lagoons in Oman: Sawadi and Qurum. Our analysis revealed that microbial communities in sediment, surface water, plastic debris, and snail guts exhibited similarities between the two lagoons, likely due to comparable environmental conditions such as temperature, salinity, total dissolved solids (TDS), and electrical conductivity. However, significant differences were noted in the snail gut microbiota, possibly due to differences in the natural versus man-made environments and varying pollution levels. In Qurum’s natural mangroves, the stable and mature ecosystem supported diverse microbial communities, while the man-made mangroves in Sawadi showed different microbial colonization patterns, influenced by higher pollution levels, including microplastics. Proteobacteria were the most abundant phylum across all substrates in both lagoons, with Gammaproteobacteria being particularly dominant. Bacteroidetes, the second most abundant phylum, played significant roles in nutrient cycling. The genus *Vibrio*, potentially including pathogenic species, was prevalent across most substrates, indicating a potential health risk in these habitats. Each substrate harbored distinct microbial communities. For instance, water samples contained unique genera such as *Sunxiuqinia* and *Pseudomonas*. Plastic surfaces, despite being artificial, support high microbial diversity, including bacteria capable of degrading plastics. Environmental conditions, substrate composition, and pollution levels were identified as major factors shaping microbial communities in the lagoons. The higher pollution levels in Sawadi supported microbial communities adapted to these conditions, whereas Qurum’s lower pollution levels favored more balanced microbial ecosystems.

The identification of distinct microbial communities among different sampling sites and different substrata in mangrove habitats indicates that environmental factors are influencing microbial communities, as microbial populations are impacted by the physical, chemical, and biological properties of the environment. Due to climate change and increasing anthropogenic pressure the microbial communities associated with mangrove forests are expected to undergo significant shifts. Yet, without the knowledge of existing microbial communities, we cannot accurately assess the extent of these changes. As mangrove habitats in the world face increasing threats, integrating microbial ecology research into ecosystem management policies becomes imperative. Recognizing the role of microorganisms in enhancing ecosystem resilience against global changes underscores the urgency of incorporating microbiome data into mangrove conservation strategies.

## Data Availability

The raw data supporting the conclusions of this article will be made available by the authors on request.

## Supplementary Materials

The following supporting information can be downloaded at: https://www.mdpi.com/article/10.3390/microorganisms12081561/s1, Figure S1: Polymer spectra, (A) HDPE, (B) PE, and (C) LDPE, for the plastics been collected in the study area; Figure S2: Transects (T1, T2, T3) within the sampling areas of the lagoons; Figure S3. Mean relative abundance of phyla across four substrates in two lagoons: sediment (A), plastic (B), water (C), and snail (D); Table S1: Study area for collecting four substrates (Snail, plastic, Water, and Sediment) from two mangrove lagoons; Table S2: Physical parameters of surface water in Sawadi and Qurum mangrove lagoons; Table S3: Diversity analysis of microbes across various substrates in Sawadi and Qurum lagoons; Table S4: Diversity Analysis of Microbes Across Various Substrates in Sawadi and Qurum Lagoons; Table S5: Descriptive statistics and Shapiro-Wallis Test results for normality of OTU distribution in Sawadi and Qurum lagoons; Table S6: Analysis of variance (ANOVA) for 16s rRNA analysis; Table S7: Normality and median equality tests for phylum relative abundance in sediments from Sawadi and Qurum lagoons; Table S8: Normality and Median Equality Tests for Phylum Relative Abundance in Water from Sawadi and Qurum Lagoons; Table S9: Normality and median equality tests for Phylum relative abundance in gut microbiota from Sawadi and Qurum lagoons; Table S10: Normality test results for Phylum relative abundance in plastic from Sawadi and Qurum lagoons; Table S11: Normality and median equality tests for class relative abundance in sediments from Sawadi and Qurum lagoons; Table S12: Comparative analysis of class relative abundance in gut microbiota from Sawadi and Qurum lagoons; Table S13: Comparative analysis of class relative abundance in water from Sawadi and Qurum lagoons; Table S14: Comparative analysis of class relative abundance in plastic from Sawadi and Qurum lagoons; Table S15: Comparative analysis of normality and median equality tests for class level across different substrates; Table S16: Comparative analysis of normality and median equality tests for genus level between Sawadi and Qurum Lagoons; Table S17: Comparative analysis of normality and median equality tests for genus relative abundance in sediment from Sawadi and Qurum lagoons; Table S18: Comparative analysis of normality and median equality Tests for genus relative abundance in water from Sawadi and Qurum lagoons; Table S19: Comparative analysis of normality and median equality tests for genus relative abundance in the gut from Sawadi and Qurum lagoons; Table S20: Comparative analysis of normality and median equality tests for genus relative abundance in plastic from Sawadi and Qurum lagoons.
