# Supplementary material for: Bacterial Communities across Multiple Ecological Niches (Water, Sediment, Plastic, and Snail Gut) in Mangrove Habitats
Source: Microorganisms. 2024 Jul 30;12(8):1561. doi: 10.3390/microorganisms12081561 (PMC11356523; doi:10.3390/microorganisms12081561)
Supplement: Supplementary file 1 [file microorganisms-12-01561-s001.zip › microorganisms-3075484-supplementary.pdf]

Supplementary Data

Supplementary Table S1. Study area for collecting four substrates (Snail, plastic, Water, and Sediment) from two mangrove lagoons in the Sea of Oman

Name of Lagoon	Coordinates	Area	Description
**Sawadi**	23°45'41.99"N 57°47' 29.64" E	Al-Batinah South	Afforested seedling of *Avicennia marina* was undertaken by the Ministry of Environment and Climate Affairs starting in 2002. The aim of this initiative was to establish a new mangrove area. The total area covered by the mangrove is approximately 23.6 hectares, and the overall wetland area, which includes other wetland habitats, spans around 177 hectares. In terms of proximity, the nearest town is located approximately 78 meters away from the edge of the lagoon associated with the mangrove. This close proximity suggests that the mangrove serves as an important natural feature in the vicinity of the local community.
**Qurum Natural Reserve**	N23°37'20.45"/E 58°28'37.34"	Muscat	The natural reserve is designated as a Ramsar location, indicating its international importance as a wetland area. The wetland itself spans approximately 90 hectares, with the mangrove habitat covering an area of about 60.9 hectares. This demonstrates the significant presence and contribution of the *Avicennia marina* mangroves within the wetland ecosystem. The wetland serves as a crucial habitat for a variety of bird species, both local and migratory. It is an attractive destination for many birds, offering a diverse range of species that rely on the wetland for various purposes. Additionally, the wetland supports a rich aquatic ecosystem, housing numerous types of fish, crustaceans, and other organisms. These details highlight the ecological importance of the wetland and its natural mangroves. The Ramsar designation signifies its global significance in terms of biodiversity conservation and the protection of wetland ecosystems. The presence of diverse bird species, along with a thriving aquatic ecosystem, further underscores the ecological value of the wetland and its role in supporting wildlife populations.

Supplementary Data

Supplementary Table S2.Physical parameters of surface water in Sawadi and Qurum mangrove lagoons

Location	Transect no	Physical Parameter	Value
Sawadi	T1	Temperature	32.1
		Water Level	-
		Electrical Conductivity	54.7
		Total Dissolved Solids	27.3
		Salinity	36.2
		Refractive index (RES)	18.30
	T2	Temperature	32.2
		Water Level	
		Electrical Conductivity	54.5
		Total Dissolved Solids	27.3
		Salinity	36.1
		Refractive index (RES)	18.40
	T3	Temperature	30.7
		Water Level	-
		Electrical Conductivity	54.2
		Total Dissolved Solids	27.1
		Salinity	35.9
		Refractive index (RES)	18.50
Qurum	T1	Temperature	30.8
		Water Level	-
		Electrical Conductivity	56.2
		Total Dissolved Solids	28.1
		Salinity	37.3
		Refractive index (RES)	17.80
	T2	Temperature	30.8
		Water Level	-
		Electrical Conductivity	56.5

		Total Dissolved Solids	28.2
		Salinity	37.6
		Refractive index (RES)	17.70
	T3	Temperature	32
		Water Level	-
		Electrical Conductivity	57.1
		Total Dissolved Solids	28.5
		Salinity	37.9
		Refractive index (RES)	17.50

Supplementary Data

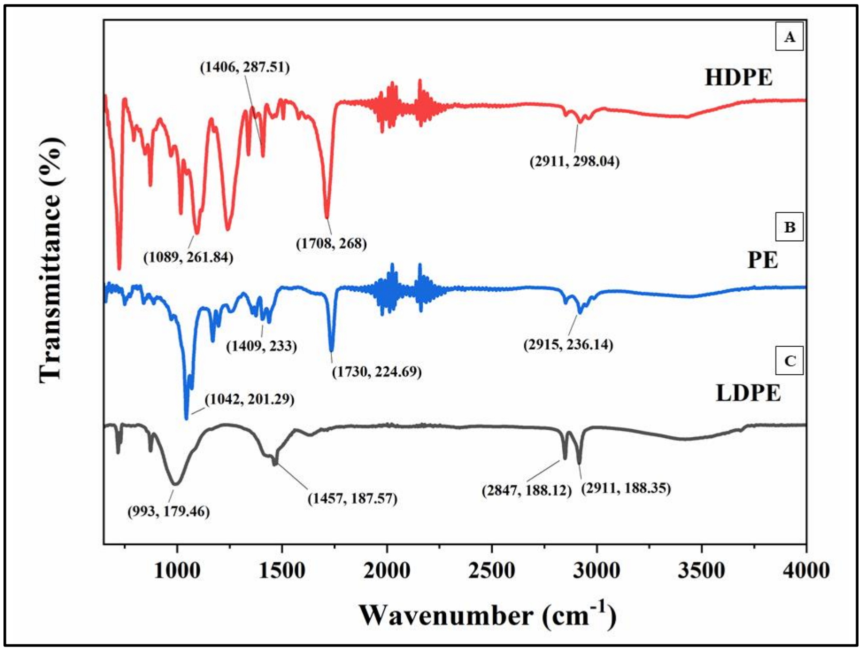

Supplementary Figure S1. Polymer spectra, (A) HDPE, (B) PE, and (C) LDPE, for the plastics been collected in the study area

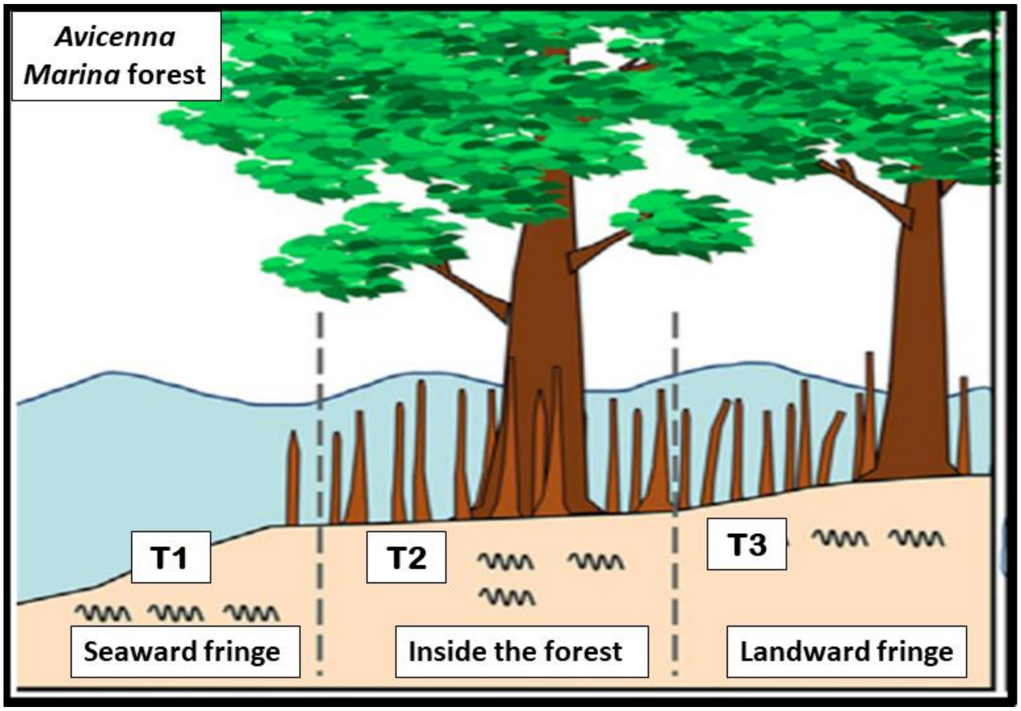

Supplementary Figure S2: Transects (T1, T2, T3) within the sampling areas of the lagoons.

Supplementary Data

Supplementary Table S3: Diversity analysis of microbes across various substrates in Sawadi and Qurum lagoons

Substrate	Location	Shannon index	Evenness index	Chao index
**Sediment**	Sawadi	3.002	0.541	15
	Qurum	2.879	0.517	20
**Water**	Sawadi	3.187	0.56	22
	Qurum	2.048	0.36	21
**Gut microbiota (Snail)**	Sawadi	0.02	0.003	3
	Qurum	0.623	0.091	10
**Plastic**	Sawadi	3.361	0.589	29
	Qurum	2.926	0.513	28

Supplementary Table S4 . Diversity Analysis of Microbes Across Various Substrates in Sawadi and Qurum Lagoons

Analysis	**Sawadi Lagoon**				**Qurum Lagoon**			
	**Sediment**	**Water**	**Gut microbiota**	**Plastic**	**Sediment**	**Water**	**Gut microbiota**	**Plastic**
Total OTUs	59560	86662	345831	216460	69016	89347	335165	195609
Mean OTUs	45.4	66	263.4	164.9	52.6	68	255.3	148.9
Standard Deviation	396.7	654.9	7788.7	2555.3	538.8	719.5	3912.3	2537.8
Total Phylum	297.7	299.9	1000	300	297.1	299.8	999.1	300
Mean Phylum	42.5	42.8	142.9	37.5	42.4	42.8	142.7	37.5
Standard Deviation	86.7	103.3	377.9	58.9	101.7	98.6	425.5	55.1
Total Class	302.4	299.9	999.95	300	304.2	299.8	994.2	300
Mean Class	21.6	17.7	142.9	18.8	21.7	17.6	90.4	18.8
Standard Deviation	56.9	51.19	377.9	33	59.9	50.4	254.2	38.9
Total Genus	253.4	296.2	998	219.9	262.1	294.7	926.8	300
Mean Genus	16.9	14.1	332.7	5.4	13.1	14	103	5.4
Standard Deviation	25.6	33.2	574.1	11.4	28	31.9	262.4	41.7

Supplementary Data

Statistical Analysis

Supplementary Table S5: Descriptive statistics and Shapiro-Wallis Test results for normality of OTU distribution in Sawadi and Qurum lagoons.

Descriptive Statistics					
	**N**	**N Missing**	**Mean**	**Standard Deviation**	**SE of Mean**
**Sawadi**	4	0	27907.775	6070.50728	3035.25364
**Qurum**	4	0	28980.625	4371.41017	2185.70509
**Normality Test**					
**Shapiro-Wilk**	**DF**	**Statistic**	**p-value**	**Decision at level(5%)**	
**Sawadi**	4	0.94473	0.68335	Can't reject normality	
**Qurum**	4	0.91719	0.52131	Can't reject normality	

Supplementary Table S6: Analysis of variance (ANOVA) for 16s rRNA analysis

One Way ANOVA					
	**DF**	**Sum of Squares**	**Mean Square**	**F Value**	**Prob>F**
Model	2	2.16E+09	1.08E+09	57.88345	<0.0001
Error	9	1.68E+08	1.87E+07		
Total	11	2.33E+09			

Fit Statistics			
**R-Square**	**Coeff Var**	**Root MSE**	**Data Mean**
0.92787	0.22775	4318.96157	18963.63333

Supplementary table S 7: Normality and median equality tests for phylum relative abundance in sediments from Sawadi and Qurum lagoons

Normality Test Results		
	**Sawadi Sediment**	**Qurum Sediment**
N	7	7
Shapiro-Wilk W	0.5638	0.4945
p-value (normal)	0.0001	1.41E-05
**Kruskal-Wallis Test Results**		
H (chi2)	0.6898	
Hc (tie corrected)	0.6913	
p-value (same)	0.4057	
Conclusion	There is no significant difference between sample medians	

Supplementary Data

Supplementary table S8: Normality and Median Equality Tests for Phylum Relative Abundance in Water from Sawadi and Qurum Lagoons

**Normality Test Results**		
	**Sawadi Water**	**Qurum Water**
N	7	7
Shapiro-Wilk W	0.4996	0.5246
p-value (normal)	1.06E-05	3.35E-05
**Kruskal-Wallis Test Results**		
H (chi2)	0.03673	
Hc (tie corrected)	0.0369	
p-value (same)	0.8477	
Conclusion	There is no significant difference between sample medians	

Supplementary Table S9: Normality and median equality tests for Phylum relative abundance in gut microbiota from Sawadi and Qurum lagoons

**Normality Test Results**		
	**Sawadi Gut**	**Qurum Gut**
N	7	7
Shapiro-Wilk W	0.453	0.524
p-value (normal)	4.14E-06	3.33E-05
**Kruskal-Wallis Test Results**		
H (chi2)	5	
Hc (tie corrected)	5	
p-value (same)	0.02535	
Conclusion	There is a significant difference between sample medians	
**Dunn Post Hoc**		
Sawadi Gut		0.02525
Qurum Gut	0.02525	

Supplementary Data

Supplementary Table S10: Normality test results for Phylum relative abundance in plastic from Sawadi and Qurum lagoons

Normality Test Results		
	**Sawadi Plastic**	**Qurum Plastic**
N	8	8
Shapiro-Wilk W	0.7614	0.7
p-value (normal)	0.01	0.002
**Kruskal-Wallis Test Results**		
H (chi2)	0.466	
Hc (tie corrected)	0.4674	
p-value (same)	0.4942	
Conclusion	There is no significant difference between sample medians	

Supplementary table S11 :Normality and median equality tests for class relative abundance in sediments from Sawadi and Qurum lagoons

Normality Test Results		
	**Sawadi Sediment**	**Qurum Sediment**
N	14	14
Shapiro-Wilk W	0.3922	0.3859
p-value (normal)	9.45E-07	8.55E-07
**Kruskal-Wallis Test**		
H (chi2)	0.076	
Hc (tie corrected)	0.07602	
p-value (same)	0.7828	
Conclusion	There is no significant difference between sample medians	

Supplementary Data

Supplementary table S12: Comparative analysis of class relative abundance in gut microbiota from Sawadi and Qurum lagoons

Normality Test Results		
	**Sawadi Gut of snail**	**Qurum Gut of snail**
**N**	10	10
**Shapiro-Wilk W**	0.3657	0.3819
**p-value (normal)**	1.00E-07	1.54E-07
**Kruskal-Wallis Test for Equal Medians**		
**H (chi2)**	9.143	
**Hc (tie corrected)**	10.05	
**p-value (same)**	0.001524	
**Conclusion**	There is a significant difference between sample medians	
**Dunn Post Hoc**		
**Sawadi Gut**	0.001524	
**Qurum Gut**		0.001524

Supplementary Table S13: Comparative analysis of class relative abundance in water from Sawadi and Qurum lagoons

Normality Test Results		
	**Sawadi Water**	**Qurum Water**
N	17	17
Shapiro-Wilk W	0.4054	0.4093
p-value (normal)	2.25E-07	2.41E-07
**Kruskal-Wallis Test for Equal Medians**		
H (chi2)	0.1569	
Hc (tie corrected)	0.157	
p-value (same)	0.6919	
Conclusion	There is no significant difference between sample medians	

Supplementary Table S14: Comparative analysis of class relative abundance in plastic from Sawadi and Qurum lagoons

Normality Test Results		
	**Sawadi Plastic**	**Qurum Plastic**
**N**	16	16
**Shapiro-Wilk W**	0.594	0.565
**p-value (normal)**	1.42E-05	7.81E-05
**Kruskal-Wallis Test for Equal Medians**		

**H (chi2)**	3.841	
**Hc (tie corrected)**	3.848	
**p-value (same)**	0.04981	
**Conclusion**	There is a significant difference between sample medians	
**Dunn Post Hoc**		
**Sawadi Plastic**	0.04981	
**Qurum Plastic**		0.04981

Supplementary Table S15: Comparative analysis of normality and median equality tests for class level across different substrates

**Normality Test for Class Level**				
	**Sediment**	**Gut**	**Water**	**Plastic**
**N**	22	22	22	20
**Shapiro-Wilk W**	0.3123	0.2552	0.3453	0.5278
**p-value (normal)**	3.43E-09	1.29E-09	6.18E-09	5.73E-07
**Kruskal-Wallis Test for Equal Medians**				
**H (chi2)**	10.95			
**Hc (tie corrected)**	11.74			
**p-value (same)**	0.008335			
**Conclusion**	There is a significant difference between sample medians			
**Dunn Post Hoc**				
**Sediment**		0.03201	0.0615	0.4431
**Gut**	0.03201		0.7833	0.0042
**Water**	0.0615	0.7833		0.0095
**Plastic**	0.4431	0.0042	0.0095	

Supplementary Table S16 : Comparative analysis of normality and median equality tests for genus level between Sawadi and Qurum Lagoons

**Descriptive Analysis**		
	**Sawadi lagoon Plastic**	**Qurum lagoon Plastic**
N Analysis	29	29
N Missing	0	0
Mean	3.44828	3.44828
Standard Deviation	5.59911	6.4736
SE of Mean	1.03973	1.20212

Supplementary Data

Shapiro-Wallis Test		
DF	29	29
Statistic	0.59906	0.60996
p-value	<0.0001	<0.0001
Decision at level (5%)	Reject normality	Reject normality
**Kruskal-Wallis Test for Equal Medians**		
H (chi2)	2.137	
Hc (tie corrected)	2.3	
p-value (same)	0.1294	
Conclusion	There is no significant difference between sample medians	

Supplementary Table S17: Comparative analysis of normality and median equality tests for genus relative abundance in sediment from Sawadi and Qurum lagoons

Normality Test Results		
	**Sawadi Sediment**	**Qurum Sediment**
N	20	20
Shapiro-Wilk W	0.5655	0.4626
p-value (normal)	1.32E-06	1.48E-07
**Kruskal-Wallis Test for Equal Medians**		
H (chi2)	0.0007317	
Hc (tie corrected)	0.0007331	
p-value (same)	0.9784	
Conclusion	There is no significant difference between sample medians	

Supplementary Table S18: Comparative analysis of normality and median equality Tests for genus relative abundance in water from Sawadi and Qurum lagoons

Normality Test Results		
	**Sawadi Water**	**Qurum Water**
N	21	21
Shapiro-Wilk W	0.4065	0.4199
p-value (normal)	3.11E-08	4.03E-08
**Kruskal-Wallis Test for Equal Medians**		
H (chi2)	0.007752	
Hc (tie corrected)	0.007752	

p-value (same)	0.9298
Conclusion	There is no significant difference between sample medians

Supplementary Table S19: Comparative analysis of normality and median equality tests for genus relative abundance in the gut from Sawadi and Qurum lagoons

**Normality Test Results**		
	**Sawadi Gut**	**Qurum Gut**
N	10	10
Shapiro-Wilk W	0.3667	0.4143
p-value (normal)	1.03E-07	3.65E-07
**Kruskal-Wallis Test for Equal Medians**		
H (chi2)	6.801	
Hc (tie corrected)	7.26	
p-value (same)	0.007051	
Conclusion	There is a significant difference between sample medians	
**Dunn Post Hoc**		
Sawadi Gut		0.00705
Qurum Gut	0.007051	

Supplementary Table S20: Comparative analysis of normality and median equality tests for genus relative abundance in plastic from Sawadi and Qurum lagoons

**Normality Test Results**		
	**Sawadi Plastic**	**Qurum Plastic**
N	29	29
Shapiro-Wilk W	0.5991	0.61
p-value (normal)	1.00E-07	1.36E-07
**Kruskal-Wallis Test for Equal Medians**		
H (chi2)	2.137	
Hc (tie corrected)	2.3	
p-value (same)	0.1294	
Conclusion	There is no significant difference between sample medians	

Supplementary Data

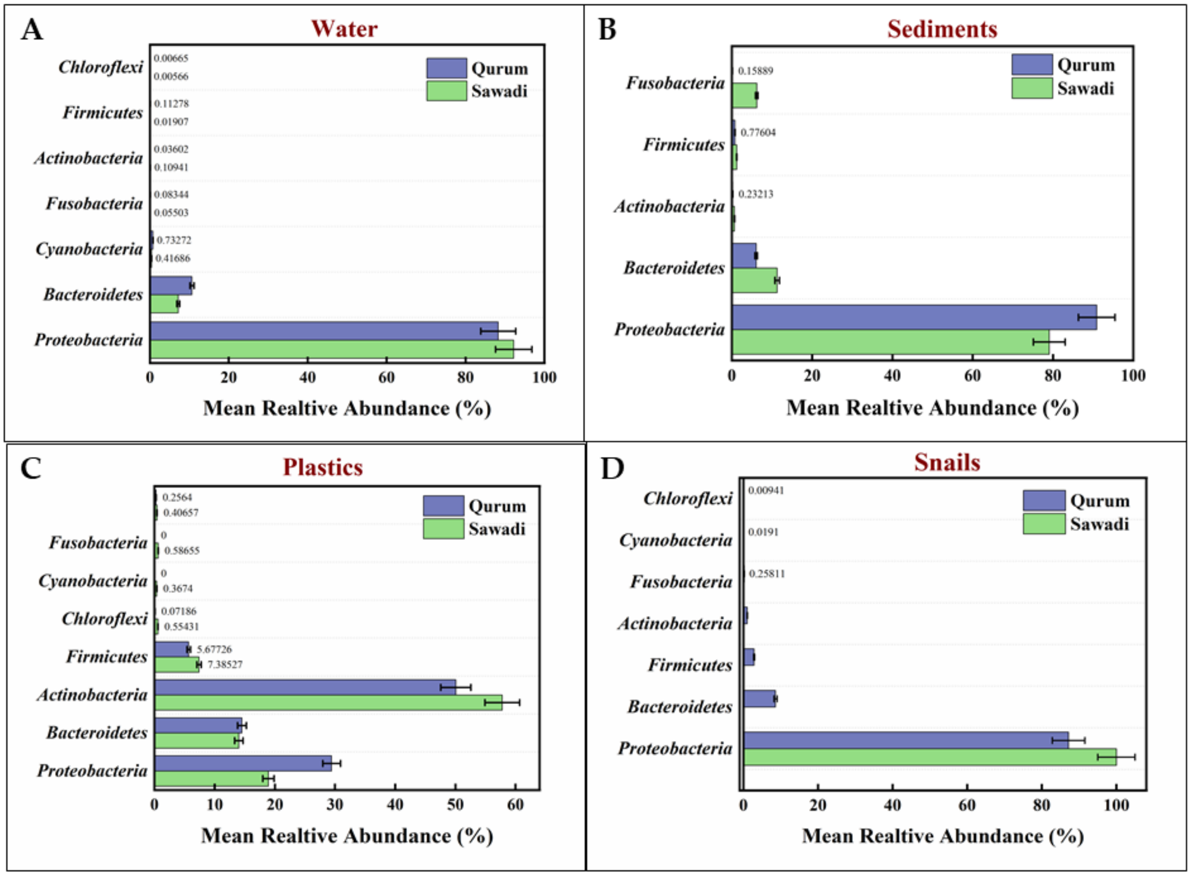

Supplementary Figure S3. Mean relative abundance of phyla across four substrates in two lagoons: sediment (A), plastic (B), water (C), and snail (D)
